# Macro-FSH is a rare cause of inappropriately high FSH concentrations

**DOI:** 10.1530/EDM-23-0144

**Published:** 2024-10-21

**Authors:** Beatrice Mantovani, Rita Indirli, Valeria Lanzi, Iulia Petria, Maura Arosio, Giovanna Mantovani, Edgardo Somigliana, Matteo Vidali, Ferruccio Ceriotti, Emanuele Ferrante

**Affiliations:** 1 Endocrinology Unit, Fondazione IRCCS Ca’ Granda Ospedale Maggiore Policlinico, Milan, Italy; 2 Department of Clinical Sciences and Community Health, University of Milan, Milan, Italy; 3 Infertility Unit, Fondazione IRCCS Ca' Granda Ospedale Maggiore Policlinico, Milan, Italy; 4 Clinical Pathology Unit, Fondazione IRCCS Ca’ Granda Ospedale Maggiore Policlinico, Milan, Italy

**Keywords:** Follicle Stimulating Hormone (FSH), Macro-FSH, laboratory interference, infertility, primary ovarian insufficiency (POI)

## Abstract

**Summary:**

Assessment of hormone concentrations can be subjected to laboratory pitfalls. Macro-hormones are hormone–autoantibody complexes which are cleared slowly from circulation and cause a false elevation in hormones’ concentrations. Macro-prolactin and macro-thyroid-stimulating hormone (TSH) are most frequently encountered while macro-follicle-stimulating hormone (FSH) has been rarely reported. We describe the case of a 30-year-old woman who had a gynaecological consultation due to failure in achieving pregnancy after 8 months of unprotected intercourse. She had regular menses, did not complain of climacteric symptoms and her medical history was unremarkable. Antral follicle count and anti-mullerian hormone concentrations were normal, and regular ovulation was documented. Unexpectedly, high early follicular phase FSH concentrations were confirmed on two occasions (57 and 51 IU/L), raising the suspicion of primary ovarian insufficiency. After excluding Turner’s syndrome and autoimmune oophoritis, a laboratory artifact was hypothesized. Following polyethylene glycol precipitation, FSH levels dropped from 41.1 IU/L to 6.54 IU/L (recovery 16%) and the presence of macro-FSH was concluded. Laboratory interference can lead to misdiagnosis and unnecessary treatments. A laboratory artifact should be suspected when inconsistency exists between clinical presentation and laboratory results. Only five other cases of macro-FSH have been reported to date. Although macro-hormones generally have low biological activity and do not require treatment, the role of anti-FSH antibodies has been hypothesized in primary ovarian insufficiency and *in vitro* fertilization failure.

**Learning points:**

- Hormone quantification is a cornerstone in the diagnostic workup of endocrine disorders, but it can be subjected to laboratory interferences which can lead to unnecessary investigations and inappropriate treatments.
- A laboratory artifact should be suspected when a discrepancy is observed between clinical presentation and laboratory results, when extremely unusual analyte concentrations are observed and when inconsistent results are obtained by different analytical methods.
- Macro-hormones are hormone–autoantibody complexes which are cleared slowly from circulation and cause a false elevation in hormone concentrations. Macro-prolactin and macro-TSH are most frequently encountered, while macro-FSH has been rarely reported.
- Macro-hormones can be detected by polyethylene glycol precipitation, gel filtration chromatography, or by using protein G or protein A columns.
- Although macro-hormones generally have low biological activity and do not require treatment, the role of anti-FSH antibodies has been hypothesized in primary ovarian insufficiency and *in vitro* fertilization failure.

## Background

Hormone quantification is a cornerstone in the diagnostic workup of endocrine disorders, but erroneous results can lead to unnecessary investigations and inappropriate treatments. Cases of misdiagnosis due to analytical problems have been reported since many factors can influence laboratory results and confound their interpretation (1).

Immunoassay is a qualitative and quantitative analytical technique used for a large variety of hormone analyses. However, many interferences in immunoassay have been described: cross-reaction, biotin, heterophile antibodies and anti-analyte antibodies (1).

In most cases, macro-hormones are complexes of hormone-directed endogenous immunoglobulins, which can alter the half-lives and physiological roles of hormones *in vivo* while preserving antigenicity or can interfere directly with the *in vitro* immunoassay. In particular, false elevation in hormone concentrations can be observed in the presence of macro-hormones (1). Several autoantibody-hormone complexes have been reported, most notably macro-prolactin (macro-PRL) and macro-TSH.

Macro-PRL, which results from the binding of immunoglobulins G (IgG) to circulating prolactin, is a large molecular size complex resulting in an inactive form with a long half-life. The prevalence of macro-PRL in studies in which all hyperprolactinaemic samples were screened for macro-PRL, is 15–46% (2). Therefore, macro-PRL constitutes a clinically relevant issue. Since the bioactivity of macro-PRL is low, patients with macro-PRL do not require treatment or further follow-up (2).

Macro-TSH is mostly composed of TSH and anti-TSH autoantibodies of the IgG class. Its prevalence is estimated to be 1.5% in patients with subclinical hypothyroidism. No thyroid hormone replacement therapy is required in patients with macro-TSH (3).

Physicians should suspect an interference in immunoassays when laboratory results are inconsistent with clinical presentation. In such cases, the first step is to repeat hormonal measurement by a different assay, while the presence of macro-hormones can be evaluated through polyethylene glycol (PEG) precipitation (4).

Herein we report a rare case of false elevation in serum FSH concentrations in a young woman due to macro-FSH. Only 4 other cases in women (4, 5, 6, 7) and 1 in men (7) have been reported to date, with varied presentations.

## Case presentation

A 30-year-old woman was referred to our Endocrinology Unit from the Infertility Unit for suspected primary ovarian insufficiency (POI). She had been having unprotected intercourse for 8 months, but pregnancy was not obtained. Therefore, the patient sought a gynaecological consultation.

Her medical history was unremarkable except for childhood-onset migraine treated with non-steroidal anti-inflammatory drugs. Menarche had occurred at the age of 14, she had normal female secondary sexual characteristics and regular menses. She did not complain of climacteric symptoms.

Preliminary blood tests revealed increased FSH levels assessed during the early follicular phase and confirmed on two occasions 1 month apart (57.5 and 51.0 IU/L, reference interval for follicular phase: 3.5–12.5; Cobas Elecysy FSH, Roche Diagnostic GmbH). Estradiol (75.5 ng/L, reference interval for follicular phase: 16.2–313) and LH (luteinizing hormone, 5.2 IU/L, reference interval for follicular phase: 2.4–12.6) levels were normal.

Transvaginal ultrasound revealed a regular appearance of the uterus and ovaries, with an endometrial thickness of 7 mm and an antral follicle count of 19 (11 + 8). Anti-mullerian hormone was normal as well (4.9 µg/L, reference interval: 2–5). Although progesterone concentrations assessed on days 21 and 23 of the menstrual cycle were low-normal (3.97 and 6.69 µg/L respectively), ultrasound follicular monitoring showed the appearance of corpus luteum between days 15 and 18, and self-monitoring with rapid urine test confirmed the occurrence of LH peak on day 15.

## Investigation

To investigate the main causes of POI, karyotype on peripheral blood lymphocytes was assessed, and autoimmune endocrine diseases were screened. A normal female karyotype was found (46,XX on 30 metaphases), and anti-ovary antibodies, as well as other associated autoimmune endocrine diseases, were not detected (Table 1).

**Table 1:** Endocrine autoimmune disease screening and anterior pituitary function assessment.

	Value	Reference range
Hemoglobin (g/dL)	12.6	12.0–16.0
Mean corpuscular volume (fL)	90.4	82.0–96.0
Platelets (10^9^/L)	232	150–400
Neutrophils (10^9^/L)	2.6	2.0–7.0
Lymphocytes (10^9^/L)	1.6	1.0–4.0
S-Glucose (mg/dL)	95	74–106
S-Creatinine (mg/dL)	0.79	0.51–0.95
S-Sodium (mmol/L)	139	135–145
S-Potassium (mmol/L)	3.8	3.5–5.0
S-Anti-ovary antibodies	<1:10	<1:10
S-Anti-parietal cell antibodies	<1:40	<1:40
S-Anti-Glutamate decarboxylase antibodies (IU/mL)	7.60	0.00–30.00
S-Antithyroglobulin antibodies (IU/mL)	<0.9	<4.0
S-Thyroid peroxidase antibodies (IU/mL)	0.6	<9.0
S-IgA (mg/dL)	220	70–400
S-Anti-tissue transglutaminase antibodies, IgA (UA/mL)	0.6	<7.0
S-TSH (mIU/L)	2.26	0.40–4.80
S-FT4 (pmol/L)	15.56	7.82–17.29
S-FT3 (pmol/L)	5.78	3.38–6.45
P-ACTH (ng/L)	24.9	5–50
S-Cortisol (µg/dL)	14.4	6.7–22.6
S-Prolactin (mIU/L)	392	70–566
S-IGF-1 (ng/mL)	150.8	57.0–305.0
S-FSH (IU/L)	46.27	3.85–8.78

ACTH, adrenocorticotropic hormone; FSH, follicle-stimulating hormone; FT3, free triiodothyronine; FT4, free thyroxine; IgA, immunoglobulin A.; IGF-1, insulin-like growth factor 1; P, plasma; S, serum; TSH, thyroid-stimulating hormone.

Even though clinical presentation was not typical, anterior pituitary hormones were assessed to exclude a gonadotroph pituitary adenoma. Gonadotroph pituitary adenomas in women generally present with ovarian hyperstimulation syndrome, high estradiol levels and high-normal FSH (8), which was not the case with our patient. Indeed, intact anterior pituitary function was confirmed in our patient.

Considering the negativity of all assessments performed so far as well as the discrepancy between biochemical and clinical findings, a false elevation in FSH concentrations was finally suspected. A new blood sample was obtained and FSH was reassessed after polyethylene glycol (PEG) precipitation. The presence of macro-FSH was assessed by precipitating 250 µL of serum treated with 250 µL of 25% PEG. The precipitable FSH (%) was calculated using the formula: (1−(2* post-PEG FSH/pre-PEG FSH)*100). Samples with a precipitable FSH >75% were considered as macro-FSH positives.

The baseline FSH concentration was 41.1 IU/L but it dropped to 6.54 IU/L after PEG precipitation (recovery 16%). Therefore, the presence of macro-FSH was concluded, and POI was ruled out.

## Outcome and follow-up

Despite the normality of the hypothalamus-pituitary-ovary axis functioning, the patient returned to the infertility clinic after 8 more months because natural conception had not occurred yet. Her partner’s semen analysis was normal. Controlled ovarian stimulation was performed, followed by homologous intrauterine insemination, and a twin pregnancy was attained.

## Discussion

Hormone assessment is frequently subject to laboratory pitfalls due to a number of causes, including cross-reaction, hook effect, biotin, heterophile antibodies and anti-analyte antibodies (1). As it can lead to misdiagnosis and unnecessary treatments, laboratory interference is a clinically relevant issue.

POI is a disorder characterized by a decline in ovarian function in women younger than 40 years of age (9). Patients generally present with irregular menstrual cycles or amenorrhea, along with other symptoms including hot flashes, night sweats, mood disorders, sleep disruption, vaginal dryness, and impaired sexual function. The finding of elevated FSH and low serum estradiol levels, confirmed on two occasions 1 month apart, is diagnostic (9). The underlying cause should be sought and concerns about fertility and hormone-replacement therapy should be promptly addressed, to prevent the long-term effects of estrogen deprivation. The most common causes of POI are genetic, autoimmune and iatrogenic; less frequently, toxic, metabolic, or infectious causes can be found. However, the vast majority of POI cases are idiopathic (39–67%) (9). In the case described herein, the patient presented an elevation of FSH levels, while estradiol and LH concentrations were normal. The patient did not report menstrual irregularities or any other complaint, and her ovarian reserve (assessed with antral follicle count and AMH) appeared normal. The most common causes of POI were screened: the finding of a normal female karyotype, and the negativity of anti-ovary antibodies, allowed us to exclude Turner syndrome and autoimmune oophoritis, respectively. Actually, current guidelines recommend testing anti-21 hydroxylase antibodies in place of anti-ovary antibodies, because of the low specificity and sensitivity of the latter (9). However, the absence of any other associated autoimmune disease led us to reasonably exclude the autoimmune cause. Before proceeding with other expensive and time-consuming analyses, we tested the hypothesis of a laboratory interference on FSH assessment, which led to a diagnosis of macro-FSH.

Macro-hormones can be screened by PEG precipitation and confirmed and better characterized through gel filtration chromatography or by using protein G or protein A columns (3). PEG is added to increase a solution concentration, reduce solutes’ solubility and induce precipitation of large molecular complexes (e.g. macro-hormones); a low percentage recovery of the unbound hormone is used for diagnosis (1). Gel filtration chromatography is used to separate molecules of different molecular sizes. Evidence of an elution profile at a higher molecular weight than expected, suggests the presence of macro-hormones (4). Protein G or protein A columns, which bind IgG or IgA respectively, are used to determine the percentage of antibody-bound hormones (5).

Only five cases of macro-FSH have been described in the literature to date (4, 5, 6, 7). Catteau and colleagues reported on a 33-year-old woman with polycystic ovary syndrome complaining of menstrual irregularities and primary infertility (4). Diagnostic work-up revealed normal estrogen, high-normal LH levels, and high antral follicle count, consistently with polycystic ovary syndrome, but unexpectedly high FSH. Macro-FSH was proven after PEG precipitation.

Chihara *et al*. described a 64-year-old woman with extremely high serum FSH concentration (713 and 548 IU/L, reference range in postmenopausal females 26–120 IU/L) (5) confirmed in several immunoassays. More than 80% of FSH was precipitated by PEG, and 89.4% was eluted at molecular weight >150 kDa using gel filtration chromatography, rather than at 36 kDa, as expected with monomeric FSH.

Guibet *et al*. reported a case of an 18-year-old woman suffering from polymenorrhea (6). Hormonal parameters showed high levels of FSH (29 and 30.9 IU/L, reference range: 0.6–12) along with normal LH, AMH and PRL levels. Pelvic ultrasound and pituitary MRI were also normal. PEG precipitation revealed the presence of macro-FSH (~60% of basal levels). The menstrual cycle later normalized spontaneously.

Finally, Webster *et al*. described two cases of macro-FSH: the first was a 24-year-old female with macro-FSH causing falsely high FSH levels which were repeatedly detected 5 months after first delivery (78.9 IU/L, reference interval: 2.9–8.4) (7), then after a miscarriage and a second natural successful pregnancy. The second case was a 16-year-old male with a history of acute lymphoblastic leukaemia treated with chemotherapy and testicular irradiation at 2 years of age. At presentation, FSH concentrations were extremely high (539 IU/L, reference interval: 1–8) with low testosterone levels (4.7 nmol/L, reference interval: 11–36) despite normal pubertal development. PEG precipitation showed low recovery; however, due to the results of gel filtration chromatography, putative heterophilic interference was supposed in the latter case (7). Characteristics of all cases of macro-FSH reported in the literature are described in Table 2, together with our case.

**Table 2:** Laboratory and clinical characteristics of macro-FSH patients reported in the literature.

Patient (Study)	Age (years), Sex	Basal FSH, IU/L	Basal LH, IU/L	FSH after PEG, IU/L	Menstrual cycle	Infertility	Pregnancy
1 (Catteau *et al*. (4))	33, F	112 (Elecsys)	18.6	13.6	O-AM (PCOS)	Yes	ND
2 (Chihara *et al*. (5))	64, F	548 (Elecsys)	ND	74.4	NA	No	Yes (S)
3 (Guibet *et al*. (6))	18, F	36.9 (Architect)	2	14.8*	P-MEN	ND	ND
4 (Webster *et al*. (7))	24, F	22.2 (DELFIA)	11.1	7.5	Regular	No	Yes (S)
5° (Webster *et al*. (7))	16, M	539 (Elecsys)	37	Recovery: 19% (DELFIA)	NA	NA	NA
6 Present case	30, F	41.1 (Elecsys)	5.2	6.54	Regular	Yes (primary)	Yes (ART)

*The Authors reported ~60% of macro-FSH after PEG precipitation; °Putative heterophilic interference (rather than FSH-autoantibodies) was supposed due to gel filtration chromatography results.

ART, assisted reproductive technology; LH, luteinizing hormone; NA, not applicable; ND, not described; O-AM, oligo-amenorrhea; PCOS, polycystic ovary syndrome; PEG, polyethylene glycol; P-MEN, polymenorrhea; S, spontaneous.

Overall, a laboratory artifact should be suspected when a discrepancy is observed between clinical presentation and laboratory results, when extremely unusual analyte concentrations are observed, and when inconsistent results are obtained from different analytical methods. In the case described herein, the patient lacked clinical, biochemical and radiological characteristics of POI. Of note is that LH concentrations were normal, while they generally increase along with FSH in primary hypogonadism, POI and menopause. Hormonal results should not be interpreted on a stand-alone basis but, rather, be weighed in relation to other findings.

Biological activities of macro-hormones are generally low (2), and they do not require medical intervention. For this reason, macro-FSH may be underdiagnosed while the prevalence of macro-FSH in patients with elevated FSH levels has never been investigated. Actually, antibodies to FSH may not be completely neutral to female reproductive health. Anti-FSH could interfere with the binding of FSH to its receptor, or form immune complexes enhancing its clearance (10). Consistently, anti-FSH antibodies have been associated with *in vitro* fertilization failure (10). Moreover, in a study on 36 patients with POI, antibodies to the β subunit of FSH were documented in 34 (11). Interestingly, an immunodominant epitope, between the amino acids 78 and 93, was identified. This region is involved in receptor binding, so anti-gonadotrophin autoimmunity may represent an interesting pathophysiological mechanism in POI.

In the case described herein, despite intact ovarian function, the patient presented 16-month unexplained primary infertility, which was successfully treated with controlled ovarian stimulation followed by intrauterine insemination. The role of anti-FSH antibodies cannot be excluded in this clinical setting.

## Declaration of interest

The authors declare that there is no conflict of interest that could be perceived as prejudicing the impartiality of the study reported.

## Funding statement

This work was supported by Ricerca Corrente funds from the Italian Ministry of Health to Fondazione IRCSS Ca'Granda Policlinico.

## Patient consent

Written informed consent for publication of her clinical details was obtained from the patient.

## Patient’s perspective

When I sought my first gynaecological consultation, I deeply hoped to realize my dream, that was to get pregnant and start a family. However, I was upset upon the finding of high FSH concentrations and suspicion of early menopause, as I was only 30 years old. Then, I was referred to the Endocrinology Unit and underwent several tests. I still remember the bruising on my arms following the blood draws. However, good news arrived: everything was normal. I felt so relieved!

Then, after several months of unsuccessful natural attempts, I decided to return to the Infertility Unit and finally, thanks to intrauterine insemination, my dream came true and today I have two lovely babies in my life.

## Author contributions

RI, VL, ES and EF conducted the diagnostic work-up and clinical follow-up. MV and FC carried out laboratory assessments. RI and BM drafted the manuscript. EF, ES, MV, FC GM and MA critically revised the manuscript. All authors read and approved the final version of the manuscript.
